# Associations of Long-Term PM2.5 Exposure and Physical Activity Levels with Metabolic Syndrome and Health-Related Quality of Life: A Cross-Sectional Study in Chiang Mai, Thailand

**DOI:** 10.3390/jcm15062241

**Published:** 2026-03-16

**Authors:** Sothida Nantakool, Busaba Chuatrakoon, Kochaphan Phirom, Cattaleeya Sittichoke, Supatcha Konghakote

**Affiliations:** 1 Integrated Neuro-Musculoskeletal, Chronic Disease, and Aging Research Engagement Center (ICARE Center), Department of Physical Therapy, Faculty of Associated Medical Sciences, Chiang Mai University, Chiang Mai 50200, Thailand; sothida.n@cmu.ac.th (S.N.); cattaleeya.sit@cmu.ac.th (C.S.); supatcha.k@cmu.ac.th (S.K.); 2 Research Institute for Health Sciences, Chiang Mai University, Chiang Mai 50200, Thailand; kochaphan.ph@cmu.ac.th

**Keywords:** metabolic syndrome, physical activity, PM2.5, climate changes, health-related quality of life

## Abstract

**Background/Objectives**: Although the adverse metabolic effects of PM2.5 and the health benefits of physical activity are well-established, evidence on whether physical activity modifies the association between PM2.5 exposure and metabolic syndrome or health-related quality of life (HRQoL) remains limited. **Methods**: This observational analytical cross-sectional study examined the modifying effect of physical activity on the associations between long-term PM2.5 exposure and metabolic syndrome and HRQoL in Chiang Mai, Thailand, and to explore these associations across physical activity levels using stratified analyses. A total of 347 participants (209 from higher PM2.5 areas and 138 from lower PM2.5 areas) were recruited in Chiang Mai between March and May 2024. Metabolic syndrome was assessed using blood tests and anthropometric measurement, while HRQoL was evaluated using the Thai version of the SF-36 questionnaire. Multivariable logistic regression was used to estimate odds ratios (ORs) and 95% confidence intervals (CIs) for metabolic syndrome. HRQoL differences were analyzed using generalized linear models with robust standard errors. Interaction between PM2.5 exposure and physical activity was assessed to examine potential effect modification. All models were adjusted for age, sex, BMI, smoking status, and educational level, with additional stratified analyses across physical activity levels. **Results**: Higher long-term PM2.5 exposure was associated with lower odds of metabolic syndrome (OR = 0.34, 95% CI: 0.14–0.83) but was not associated with HRQoL. Physical activity was not independently associated with either outcome, and no interaction between PM2.5 exposure and physical activity was observed. In stratified analyses, the inverse association between PM2.5 exposure and metabolic syndrome was observed only among individuals with high physical activity, while significantly lower HRQoL scores were observed among those with moderate and high physical activity levels. **Conclusions**: Higher long-term PM2.5 exposure was associated with lower odds of metabolic syndrome and lower HRQoL. Physical activity was not independently associated with these outcomes, and no interaction between PM2.5 exposure and physical activity was observed. Stratified analyses suggested variation in these associations across physical activity levels.

## 1. Introduction

Metabolic syndrome is a cluster of conditions that significantly contribute to public health burdens, particularly type II diabetes, cerebrovascular disease, and cardiovascular disease [1]. Notably, cerebrovascular and cardiovascular diseases are among the leading causes of death globally [2]. In addition, metabolic syndrome has been linked to decreased health-related quality of life (HRQoL) [3,4]. In Thailand, prevalence of metabolic syndrome has risen significantly [5,6]. Therefore, reducing the prevalence of metabolic syndrome may help lower the risk of major non-communicable diseases and improve overall well-being.

Ambient fine particulate matter (PM2.5) has been recognized as a major environmental health concern worldwide [7]. The World Health Organization (WHO) reports that 99% of the global population lives in areas where PM2.5 levels exceed the recommended limits, and that 89% of premature deaths are associated with exposure to air pollution [8]. In Thailand, particularly in the northern region, PM2.5 concentrations frequently exceed the WHO guidelines by 3–10 times during peak seasons [9]. Long-term exposure has been associated with elevated blood glucose levels and lipid abnormalities [10,11].

Conversely, physical activity is well-documented to reduce the risk of metabolic syndrome, type II diabetes, and cardiovascular diseases [12]. A meta-analysis has provided synthesized evidence that individuals with high levels of leisure-time physical activity have a 20% lower risk of developing metabolic syndrome [13]. Furthermore, the physically demanding activities of outdoor occupations have been linked to a favorable metabolic profile [14].

Although the adverse metabolic effects of PM2.5 and the protective benefits of physical activity are individually well-documented, most existing studies have examined these factors independently. Limited evidence has explored whether physical activity modifies the associations between PM2.5 exposure and metabolic syndrome and HRQoL, particularly in regions with chronically higher pollution levels. In highly polluted Southeast Asian settings such as northern Thailand, where population-level exposure is persistently elevated, it remains unclear whether the metabolic benefits of physical activity are preserved, attenuated, or operate independently of pollution-related risks.

Therefore, the primary objective was to examine whether physical activity modifies the associations between long-term PM2.5 exposure and metabolic syndrome and HRQoL in Chiang Mai, Thailand. As a secondary objective, we conducted stratified analyses across physical activity levels to explore patterns of association within different physical activity categories.

## 2. Materials and Methods

### 2.1. Study Design and Participants

This study was an observational analytical cross-sectional study that included 347 adults recruited between 27 March 2024 and 10 May 2024. Participants were recruited from multiple sites in the Mae Chaem district, Omkoi district, and San Pa Tong district, Chiang Mai Province, Thailand. Inclusion criteria were: (1) aged ≥20 years, (2) permanent residence in the study area for ≥5 years, and (3) both sexes. Exclusion criteria included: (1) unstable cardiovascular conditions within the past year, (2) unstable chronic diseases (e.g., uncontrolled diabetes mellitus or exacerbated chronic obstructive pulmonary disease) within the past year, and (3) pregnancy. All participants provided written informed consent before data collection. The study was conducted in accordance with the Declaration of Helsinki and approved by the Research Ethics Committee of the Research Institute for Health Sciences, Chiang Mai University (Approval No. 72/2023).

### 2.2. Sample Size Estimates

A target sample size was determined a priori based on feasibility, available funding, and the aim to obtain adequate representation across study sites rather than a formal statistical power calculation. An initial recruitment plan included four districts, with an approximate target of 150 participants per district. Due to budget limitations, this plan was revised prior to recruitment to include three districts only (Omkoi, Mae Jam, and San Pa Tong), with a revised target of approximately 100 participants per district (total target n = 300). Ultimately, 347 participants were recruited (Omkoi n = 112, Mae Jam n = 97, San Pa Tong n = 138), representing the maximum achievable sample within the study period and available resources.

### 2.3. Sampling Technique

Purposive sampling, a non-probabilistic sampling method, was employed to identify study areas with contrasting ambient PM2.5 exposure levels in Chiang Mai Province, Thailand. District-level exposure classification (higher vs. lower PM2.5) was defined based on the average 24-h PM2.5 concentrations recorded over the past five years. This area-based classification was selected to reflect sustained community-level exposure differences rather than short-term individual fluctuations, allowing for a comparison between chronically higher- and lower-exposure environments.

Two districts with consistently high PM2.5 accumulation, Omkoi and Mae Jam, were selected to represent higher-exposure environments. In contrast, San Pa Tong was selected as the lower-exposure comparison district, as its long-term average PM2.5 concentrations remained below the study-defined threshold of 50 µg/m^3^. This threshold corresponds to Thailand’s national 24-h ambient air quality standard for PM2.5 and was therefore used to distinguish lower- from higher-exposure areas, reflecting categories associated with potential health impacts [15].

Within each district, one subdistrict was randomly selected using simple random sampling. Subsequently, study units within the selected subdistricts were recruited through purposive sampling based on predetermined eligibility criteria.

### 2.4. Definition and Measurement of Metabolic Syndrome

Metabolic syndrome was defined as the presence of at least three of the following five components according to the National Cholesterol Education Program (NCEP) Adult Treatment Panel III (ATP III) criteria [16]: increased waist circumference (WC) (≥90 cm for men and ≥80 cm for women) [17], elevated blood pressure (≥130/85 mmHg), elevated hemoglobin A1c (≥5.7%), low high-density lipoprotein cholesterol (<40 mg/dL for men and <50 mg/dL for women), and elevated triglycerides (≥150 mg/dL). WC was measured in a horizontal plane at the midpoint between the lowest rib and the iliac crest [18]. Measurements were taken twice, and the average value was recorded if the difference between the two measurements was <1 cm; otherwise, the measurement was repeated. A 5-mL fasting blood sample was collected in the morning after at least 10 h of fasting by a professional medical technologist.

### 2.5. Physical Activity Assessment

Physical activity (PA) was assessed using the Global Physical Activity Questionnaire version 2 (GPAQ) [19]. The GPAQ includes questions on occupational, transport, and leisure-time physical activity. Total physical activity was expressed as metabolic equivalent minutes per week (MET-min/week), calculated based on the frequency and duration of activities performed during a typical week. PA levels were classified as high (≥3000 MET-min/week from any combination of walking, moderate-, or vigorous-intensity activities, or ≥1500 MET-min/week from vigorous-intensity activity), moderate (≥600 MET-min/week but not meeting the criteria for high PA), or low (not meeting the criteria for either moderate or high PA).

### 2.6. Health-Related Quality of Life

Health-related quality of life (HRQoL) was assessed using the Thai version of the 36-Item Short Form Health Survey (SF-36) questionnaire [20]. The questionnaire consists of 36 items covering eight domains: physical functioning, role limitations due to physical problems, bodily pain, general health perceptions, vitality (energy/fatigue), social functioning, role limitations due to emotional problems, and mental health (psychological distress and psychological well-being). Higher scores indicate better HRQoL.

### 2.7. Statistical Analyses

Data were analyzed using SPSS Statistics software (version 17.0, IBM Corp., Armonk, NY, USA) for windows. Data normality was assessed using the Kolmogorov–Smirnov test. For baseline characteristics, continuous data were analyzed using the independent *t*-test (age, weight, height, BMI), while dichotomous and categorical data were calculated using the chi-square test or Fisher’s exact test.

Odds ratios (ORs) and 95% confidence intervals (95% CIs) for metabolic syndrome were estimated using multivariable logistic regression models. To examine whether physical activity modified the association between long-term PM2.5 exposure and metabolic syndrome, an interaction term between PM2.5 exposure and physical activity level was included in the fully adjusted model. Covariates included age, sex, BMI, smoking status, and educational level.

Between-group differences in HRQoL scores were analyzed using generalized linear models with robust standard errors. Interaction terms between PM2.5 exposure and physical activity were included to assess potential effect modification. Models were adjusted for age, sex, BMI, smoking status, and educational level.

As a secondary analysis, stratified regression models were employed across physical activity levels to explore patterns of association between PM2.5 exposure and metabolic syndrome and HRQoL within each physical activity category. Within each physical activity category, ORs and 95% CI for metabolic syndrome associated with PM2.5 exposure were estimated using multivariable logistic regression models adjusted for age, sex, BMI, smoking status, and educational level. Similarly, stratified generalized linear models were used to assess differences in HRQoL scores associated with PM2.5 exposure within each physical activity level, adjusting for age, sex, BMI, smoking status, and educational level. Multicollinearity among covariates was assessed using variance inflation factors (VIFs). A *p*-value of <0.05 was considered statistically significant.

## 3. Results

Characteristics of 347 participants were analyzed in this study, including 209 participants in the higher PM2.5 area group and 138 participants in the lower PM2.5 area group. Participants in the higher PM2.5 area group were significantly younger, had higher weight and BMI, attained higher educational level, and demonstrated higher levels of physical activity compared to those in the lower PM2.5 area group (*p* < 0.05 for all). Regarding air pollution data, both PM2.5 concentration and the number of hotspots were significantly higher in the higher PM2.5 area compared to the lower PM2.5 area (*p* < 0.05 for all) (Table 1).

### 3.1. Association of PM2.5 Exposure and Physical Activity with Metabolic Syndrome and HRQoL

After adjustment (adjusted for age, sex, BMI, smoking status, and educational level), higher long-term PM2.5 exposure was associated with significantly lower odds of metabolic syndrome (OR = 0.34, 95% CI: 0.14 to 0.83; *p* = 0.018). However, long-term PM2.5 exposure was not significantly associated with total SF-36 score (β = −0.44, 95% CI: −2.64 to 1.76; *p* = 0.69) (Table 2).

Physical activity level was not independently associated with metabolic syndrome or HRQoL in the fully adjusted model. Furthermore, interaction terms between PM2.5 exposure and physical activity were not statistically significant for either metabolic syndrome (moderate vs. low PA: OR = 1.98, 95% CI: 0.55 to 7.19; high vs. low PA: OR = 0.47, 95% CI: 0.13 to 1.76) or total SF-36 score (all *p* > 0.05), indicating no evidence of effect modification (Table 2).

### 3.2. Stepwise-Adjusted Regression Models

In hierarchical regression analyses, the inverse association between higher PM2.5 exposure and metabolic syndrome remained statistically significant across progressively adjusted models. In Model 1 (adjusted for age and sex), higher PM2.5 exposure was associated with lower odds of metabolic syndrome (OR = 0.51, 95% CI: 0.28 to 0.92; *p* = 0.025). The association became stronger after additional adjustment for BMI and smoking (Model 2: OR = 0.32, 95% CI: 0.17 to 0.62; *p* = 0.001) and remained stable after further adjustment for educational level (Model 3: OR = 0.32, 95% CI: 0.16 to 0.62; *p* = 0.001) (Table 3).

Similarly, higher PM2.5 exposure was consistently associated with lower total SF-36 scores across all models. In the fully adjusted model (Model 3), participants in higher PM2.5 areas had a 6.24-point lower HRQoL score compared with those in lower-exposure areas (β = −6.24, 95% CI: −9.04 to −3.44; *p* < 0.001) (Table 3).

### 3.3. Stratified Analyses Across Physical Activity Level

In stratified analyses adjusted for age, sex, BMI, smoking status, and educational level, the association between PM2.5 exposure and outcomes varied across physical activity levels.

For metabolic syndrome, higher PM2.5 exposure was significantly associated with lower odds of metabolic syndrome among individuals with high physical activity (OR = 0.12, 95% CI: 0.03 to 0.45; *p* = 0.002), but not among those with low or moderate physical activity (Table 4).

For HRQoL, higher PM2.5 exposure was associated with significantly lower total SF-36 scores among participants with moderate (β = −6.62, *p* = 0.01) and high physical activity (β = −11.05, *p* < 0.001), but not among those with low physical activity. In domain-specific analyses, significant reductions in the Physical Component Summary (PCS) were observed in moderate and high physical activity groups, while a significant reduction in the Mental Component Summary (MCS) was observed only among individuals with high physical activity (β = −3.23, 95% CI: −6.34 to −0.06; *p* = 0.046) (Table 4).

## 4. Discussion

Aims of the study were to examine whether physical activity modifies the associations between long-term PM2.5 exposure and metabolic syndrome and HRQoL in Chiang Mai, Thailand, and to conduct stratified analyses across physical activity levels to explore patterns of association within different physical activity categories. This study yielded several key findings. First, higher long-term PM2.5 exposure was associated with lower odds of metabolic syndrome. Second, higher long-term PM2.5 exposure was associated with lower HRQoL scores in stepwise-adjusted models. Third, physical activity level was not independently associated with metabolic syndrome or HRQoL. Fourth, no significant interaction between long-term PM2.5 exposure and physical activity was observed. Last, stratified analyses suggest that the association between long-term PM2.5 exposure and outcomes remained observable across physical activity levels.

Interestingly, the current study found that higher long-term PM2.5 exposure was associated with lower odds of metabolic syndrome, a finding that contrasts with several previous studies reporting detrimental metabolic effects of air pollution [10,11]. One possible explanation for this unexpected association may relate to contextual differences between areas with varying pollution levels. In the present study setting, areas with higher PM2.5 exposure were predominantly rural, whereas areas with lower exposure were more urbanized. Previous studies have reported that metabolic syndrome prevalence tends to be higher in urban populations than in rural populations, potentially due to differences in lifestyle patterns, dietary behaviors, occupational physical activity, and built environments [21,22]. Rural populations may engage in higher occupational physical activity due to agricultural or manual labor and may exhibit lower levels of sedentary behavior compared with urban residents. These differences in habitual patterns may partly offset metabolic risk despite higher ambient pollution levels [23]. In the present study, this contextual pattern was also observed. Among participants with high physical activity levels, those residing in higher PM2.5 areas more frequently reported occupational-related activities (81.5%), whereas those living in lower PM2.5 areas more commonly reported leisure-time activities (29.3%). These findings suggest that the high physical activity observed in the higher PM2.5 areas may largely reflect work-related activities rather than leisure-time exercise, which may partly explain the lower odds of metabolic syndrome observed in this population.

Although several potential determinants of metabolic profiles were adjusted for in the present analysis, other important factors—including behavioral, contextual, and socioeconomic characteristics—were not captured. Previous studies have identified behavioral factors such as higher alcohol consumption and unhealthy dietary patterns as significant contributors to metabolic syndrome [24,25]. Furthermore, the current study areas were selected based on differences in long-term PM2.5 levels; the rural–urban contrast between the two settings occurred unintentionally. Consequently, differences in area-level characteristics, including occupational activity patterns and lifestyle behaviors, may introduce residual confounding and influence the observed association between long-term PM2.5 exposure and metabolic syndrome. In addition, an average age difference of approximately 15 years was observed between the exposure groups. Age is a well-established determinant of cardiometabolic risk, and older individuals are more likely to develop metabolic abnormalities. Although age was adjusted for in the statistical models, residual confounding related to differences in age distribution cannot be entirely excluded.

In addition to the association with metabolic syndrome, the present study also found that higher long-term PM2.5 exposure was associated with lower HRQoL scores. Although the interaction model did not show a significant association between long-term PM2.5 exposure and HRQoL, the stepwise adjusted models revealed a negative association. This finding suggests that demographic, behavioral, and socioeconomic covariates, including age, sex, BMI, smoking status, and educational level, may confound the relationship between PM2.5 exposure and HRQoL. After accounting for these factors, the adverse association between long-term PM2.5 exposure and HRQoL became more apparent.

PM2.5 has been recognized as a major global environmental health concern, with several studies reporting its association with metabolic syndrome [7]. Long-term exposure to PM2.5 has been linked to increased cardiometabolic risk through mechanisms such as hypertension and dyslipidemia [10,11]. In contrast, physical activity is widely recognized as a protective factor for metabolic health and has been shown to reduce the risk of metabolic syndrome [12]. However, whether physical activity modifies the association between PM2.5 exposure and metabolic syndrome remains unclear. While previous studies have largely examined the independent effects of either PM2.5 or physical activity on metabolic outcomes [10,11,12], evidence regarding their combined influence is limited. In the present study, physical activity was not independently associated with metabolic syndrome, and no statistically significant interaction between PM2.5 exposure and physical activity was observed.

Nevertheless, stratified analyses suggested that the inverse association between PM2.5 exposure and metabolic syndrome was primarily observed among individuals with higher levels of physical activity, whereas no clear association was observed in the low or moderate physical activity groups. This pattern may reflect contextual lifestyle differences between areas with varying pollution levels rather than a direct protective effect of PM2.5 exposure. In the present study setting, areas with higher PM2.5 concentrations were predominantly rural, where higher levels of physical activity may be related to occupational activities such as agricultural or manual labor. Such habitual activity patterns may contribute to lower cardiometabolic risk despite higher ambient pollution levels. Therefore, the observed association may partly reflect differences in occupational physical activity and lifestyle context between areas rather than a direct effect of air pollution.

With respect to physiological mechanisms, exposure to PM2.5 has been proposed to influence cardiometabolic health through increased oxidative stress, systemic inflammation, and endothelial dysfunction [26,27,28]. Conversely, physical activity has been shown to improve insulin sensitivity, lipid metabolism, and vascular function, all of which contribute to cardiometabolic risk reduction [29,30]. However, the present findings suggest that physical activity did not independently explain differences in metabolic outcomes in this population. Instead, the stratified patterns observed across physical activity levels may reflect differences in habitual activity patterns and environmental contexts between rural and urban settings.

Regarding health-related quality of life (HRQoL), substantial evidence has demonstrated that long-term exposure to PM2.5 is associated with poorer HRQoL in adults [31,32,33,34], whereas physical activity is generally linked to improved HRQoL [35,36]. In the present study, the interaction model did not show a significant association between PM2.5 exposure and HRQoL. However, stepwise adjusted models revealed a negative association between long-term PM2.5 exposure and HRQoL after accounting for demographic, behavioral, and socioeconomic covariates. These findings suggest that individual characteristics may influence the relationship between environmental exposure and perceived health status. Moreover, engaging in physical activity in polluted environments may increase perceived discomfort, respiratory strain, and environmental stress, which may negatively affect HRQoL regardless of baseline health status [37,38]. In addition, in rural settings, higher physical activity may largely reflect occupational or labor-related activities rather than leisure-time exercise. Such physically demanding activities, particularly when performed under unfavorable environmental conditions such as poor air quality, may contribute to physical fatigue and perceived health burden, which may negatively influence HRQoL [39].

The lower HRQoL observed among individuals engaging in moderate-to-high physical activity in areas with higher PM2.5 exposure may also reflect differences in the context and type of physical activity. Previous global surveillance studies have shown that in rural settings, a substantial proportion of physical activity may be derived from occupational or labor-intensive tasks rather than leisure-time exercise [40]. Occupational physical activity often involves prolonged physical demands and limited recovery time, which may contribute to physical fatigue and perceived burden rather than improvements in perceived quality of life. Consistent with this explanation, our findings showed that among participants with high physical activity levels, those residing in higher PM2.5 areas more commonly reported occupational-related activities, whereas those in lower PM2.5 areas more frequently reported leisure-time activities. These contextual differences highlight that the health implications of physical activity may vary depending on the type and environmental conditions in which the activity occurs.

Although this study does not allow for causal inference, the findings may still have clinical and public health relevance. Metabolic syndrome is a well-established cardiometabolic risk marker associated with increased risk of cardiovascular disease, type 2 diabetes, and premature mortality [1,2]. Therefore, identifying environmental and lifestyle factors associated with metabolic syndrome may contribute to improved population-level risk assessment. In addition, the observed associations between long-term PM2.5 exposure, physical activity, and HRQoL provide insights into how environmental and behavioral contexts may shape metabolic health and perceived well-being in communities experiencing chronic air pollution. These findings also highlight the potential importance of community-based strategies that promote safe and appropriate physical activity. Such approaches may involve collaboration across public health, rehabilitation, and community health disciplines to support metabolic health and functional well-being at the population level. This perspective may help inform future interdisciplinary efforts that integrate environmental health, lifestyle behaviors, and community-based health promotion strategies.

Several limitations of this study should be acknowledged. First, cross-sectional study design limits causal inference between long-term PM2.5 exposure, physical activity, and outcome measures (i.e., metabolic syndrome and HRQoL). Although associations were observed, the temporal sequence between exposure and outcomes cannot be determined. Future prospective longitudinal studies are required to clarify relationships and to better understand the role of physical activity on population exposed to long-term air pollution. Second, several potential residual confounding factors that may influence metabolic health and HRQoL were not assessed in the present study. These may include lifestyle and contextual factors such as dietary patterns, occupational characteristics, sleep behaviors, indoor air pollution, and healthcare accessibility. Such factors may vary across geographic settings and could partially explain the observed associations between long-term PM2.5 exposure and health outcomes. In particular, differences in lifestyle patterns and environmental contexts between study areas may introduce residual confounding that was not fully captured in the analysis. Further studies incorporating these contextual and lifestyle factors may provide a more comprehensive understanding of the combined and independent influences on metabolic syndrome and HRQoL in this population. In addition, an age difference between exposure groups was observed. This may partly reflect the non-probability sampling approach used to select study areas with different levels of PM2.5 exposure. Although age was adjusted in the statistical models, differences in age distribution between areas may still introduce residual confounding. Third, this study was conducted in areas with a moderate level of PM2.5 exposure, which may limit the generalizability of the findings to populations residing in regions with substantially higher pollution levels. Fourth, the sample size was determined based on feasibility and available resources rather than a formal statistical power calculation, which may limit statistical power to detect smaller associations. Fifth, long-term PM2.5 exposure was assigned using district-level 5-year averages, and individual-level exposure variability (e.g., time spent indoors, occupational exposure, and daily mobility) was not captured. This may lead to exposure misclassification. If largely non-differential, such misclassification would tend to attenuate associations toward the null, although differential misclassification related to health status or socioeconomic factors cannot be ruled out. Finally, physical activity was assessed using self-reported measures, which may be subject to recall bias and misclassification. Future research employing objective assessment tools, such as accelerometers, would help improve the accuracy of physical activity measurement and strengthen the validity of the findings.

## 5. Conclusions

This study concludes that higher long-term PM2.5 exposure was associated with lower odds of metabolic syndrome and lower HRQoL scores in the study population. Physical activity was not independently associated with metabolic syndrome or HRQoL, and no significant interaction between PM2.5 exposure and physical activity was observed. However, stratified analyses suggested that the association between PM2.5 exposure and health outcomes remained observable across physical activity levels. These findings highlight the potential influence of contextual and lifestyle factors in shaping metabolic syndrome and quality of life in populations exposed to long-term air pollution.

## Figures and Tables

**Table 1:** Baseline and area-based characteristics.

Variables		Higher PM2.5 Area Group (n = 209)	Lower PM2.5 Area Group (n = 138)	*p*-Value
Age (year)		44.6 ± 11.0	60.0 ± 7.9	0.000 *
Sex (male, %)		60 (64.5)	33 (35.48)	0.324
Weight (kg)		62.3 ± 11.7	59.5 ± 11.3	0.029
Height (cm)		155.9 ± 8.0	156.4 ± 7.5	0.596
BMI (kg/m^2^)		25.7 ± 4.7	24.3 ± 3.8	0.015
Educational level	Illiteracy	8 (72.7)	3 (27.27)	0.536
	≤6 years	72 (49.3)	74 (50.7)	0.000 *
	>6 years	128 (67.7)	61 (32.28)	0.002 *
Smoking history (%)	Yes	34 (64.2)	19 (35.9)	0.564
	Ex-smoker	15 (68.2)	7 (31.8)	
	No	160 (58.8)	112 (41.2)	
Physical activity level (%)	Low	87 (55.06)	71 (44.94)	0.072
	Occupational-related	16 (55.2)	13 (44.8)	
	Leisure-time-related	27 (65.9)	14 (34.1)	
	Transportation-related	87 (55.1)	73 (52.5)	
	Moderate	52 (58.43)	37 (41.57)	0.661
	Occupational-related	29 (69.0)	13 (31.0)	
	Leisure-time-related	19 (55.9)	15 (44.1)	
	Transportation-related	51 (58.6)	36 (41.4)	
	High	71 (70.30)	30 (29.70)	0.014 *
	Occupational-related	75 (81.5)	17 (18.5)	
	Leisure-time-related	29 (70.7)	12 (29.3)	
	Transportation-related	71 (44.9)	30 (21.6)	
Air Quality Index	PM2.5 concentration (µg/m^3^)	53.2 ± 6.54	24.50 ± 4.39	0.0003 *
	Hotspots (spot)	2723.75 ± 1800.76	15.5 ± 5.07	0.02 *

Continuous data are presented as mean ± standard deviation. cm, centimeter; kg, kilogram; kg/m^2^, kilogram per square meter; µg/m^3^, microgram per cubic meter; %, percentage; *, statistical significance.

**Table 2:** Association between PM2.5 exposure, physical activity, and their interaction with metabolic syndrome and HRQoL.

Variables	OR (95% CI) MetS	*p*-Value	ß (95% CI) Total SF-36	*p*-Value
PM2.5 (higher vs. lower)	0.34 (0.14 to 0.83)	0.018 *	−0.44 (−2.64 to 1.76)	0.69
PA (moderate vs. low)	0.56 (0.23 to 1.37)	0.20	1.32 (−0.96 to 3.61)	0.26
PA (high vs. low)	1.49 (0.59 to 3.76)	0.40	0.80 (−1.28 to 2.88)	0.45
PM2.5 × PA (moderate vs. low)	1.98 (0.55 to 7.19)	0.30	0.17 (−2.97 to 3.30)	0.92
PM2.5 × PA (high vs. low)	0.47 (0.13 to 1.76)	0.27	−2.10 (−5.11 to 0.91)	0.17

Adjusted by age, sex, BMI, smoking, and educational level. *, statistical significance; ß, adjusted mean difference; CI, confidence interval; SF-36, 36-item short form survey; MetS, metabolic syndrome; PA, physical activity; PM2.5, fine particulate matter.

**Table 3:** Stepwise-adjusted regression models for the associations between long-term PM2.5 exposure and metabolic syndrome and HRQoL.

Models	OR (95% CI) MetS	*p*-Value	ß (95% CI) Total SF-36	*p*-Value
Model 1	0.51 (0.28 to 0.92)	0.025 *	−6.49 (−9.28 to −3.70)	<0.001 *
Model 2	0.32 (0.17 to 0.62)	0.001 *	−6.09 (−8.93 to −3.24)	<0.001 *
Model 3	0.32 (0.16 to 0.62)	0.001 *	−6.24 (−9.04 to −3.44)	<0.001 *

*, statistical significance; ß, adjusted mean difference; CI, confidence interval; MetS, metabolic syndrome; OR, odds ratio; PM2.5, fine particulate matter; SF-36, 36-item short form survey. Lower PM2.5 was used as the reference category. Model 1: adjusted by age, sex. Model 2: adjusted by age, sex, BMI, and smoking. Model 3: adjusted by age, sex, BMI, smoking, and educational level.

**Table 4:** Stratified associations between long-term PM2.5 exposure and outcomes across physical activity levels.

PA Level	OR (95% CI) MetS	*p*-Value	ß (95% CI) Total SF-36	*p*-Value	ß (95% CI) PCS	*p*-Value	ß (95% CI) MCS	*p*-Value
Low PA	0.42 (0.45 to 1.18)	0.10	−3.21 (−7.16 to 0.74)	0.11	−3.35 (−6.21 to −0.49)	0.02 *	−0.19 (−2.62 to 2.23)	0.88
Moderate PA	0.67 (0.17 to 2.72)	0.58	−6.62 (−11.64 to −1.60)	0.01 *	−5.98 (−10.40 to −1.57)	0.008 *	0.08 (−3.05 to 3.20)	0.96
High PA	0.12 (0.03 to 0.45)	0.002 *	−11.05 (−16.41 to −5.68)	<0.001 *	−6.59 (−10.38 to −2.80)	0.001 *	−3.23 (−6.34 to −0.06)	0.046 *

Adjusted by age, sex, BMI, smoking, and educational level. Lower PM2.5 was used as the reference category. *, statistical significance; ß, adjusted mean difference; CI, confidence interval; MCS, mental component summary; MetS, metabolic syndrome; PA, physical activity; PCS, physical component summary; PM2.5, fine particulate matter; SF-36, 36-item short form survey.

## Data Availability

The data presented in this study are available upon request from the corresponding authors.

## Abbreviations

The following abbreviations are used in this manuscript:

ATP III	Adult Treatment Panel III
BMI	Body mass index
cm	centimeter
CI	Confidence interval
GPAQ	Global Physical Activity Questionnaire
HRQoL	Health-related quality of life
kg	Kilogram
kg/m^2^	Kilogram per square meter
MCS	Mental component summary
MET-minute	Metabolic equivalent in a minute
mg/dL	Milligram per deciliter
OR	Odds ratio
PA	Physical activity
PCS	Physical component summary
PM2.5	Ambient fine particular matter
NCEP	National Cholesterol Education Program
SF-36	36-Item Short Form Health Survey
wc	Waist circumference
WHO	World Health Organization
µg/m^3^	Micrograms per cubic meter
